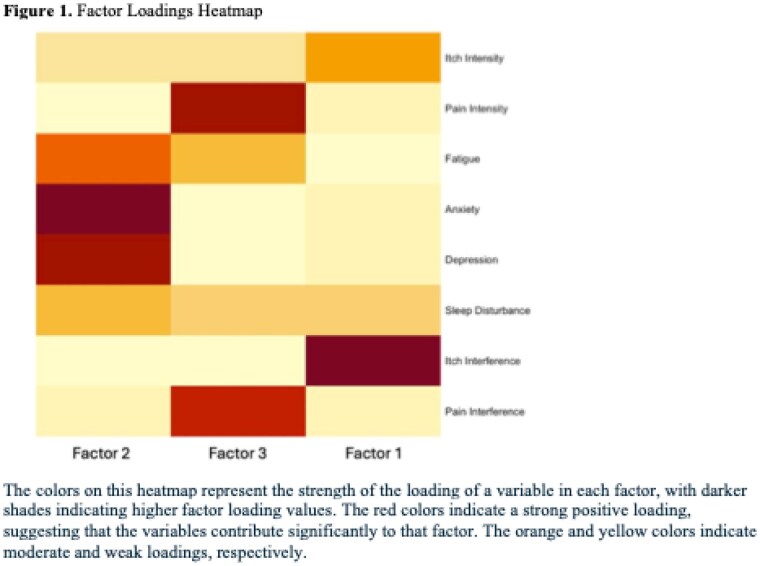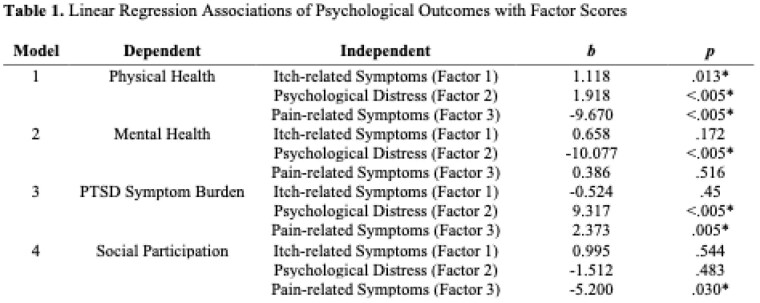# 45 Pain, Itch, and Psychological Distress Symptom Clusters in Burn Survivors: A Factor Analysis

**DOI:** 10.1093/jbcr/iraf019.045

**Published:** 2025-04-01

**Authors:** Lynn Nakad, Arushi Biswas, Sheera Lerman, Julie Caffrey, Carrie Cox, Jeannie Leoutsakos, Rachel Kornhaber

**Affiliations:** Johns Hopkins University School of Medicine; Johns Hopkins University School of Medicine; Johns Hopkins University School of Medicine; Johns Hopkins University School of Medicine; Johns Hopkins Bayview Medical Center; Johns Hopkins University School of Medicine; Charles Sturt University

## Abstract

**Introduction:**

Burn survivors experience a host of distressing and co-occurring symptoms. Identifying symptom clusters can help facilitate interventions to help improve symptom management. The purpose of this study was to identify symptom clusters among burn survivors based on the severity of prevalent chronic symptoms (pain, itch, fatigue, sleep disturbance, anxiety, and depression) and to examine their associations with psychosocial outcomes.

**Methods:**

Data were analyzed from the National Institute on Disability and Rehabilitation Research Burn Model System database. Exploratory factor analysis (EFA) was conducted on symptoms at 6 months post-burn assessed using the PROMIS-29, PROMIS Global v1.2, and 5-D itch scale. Principal components were extracted and EFA with Maximum Likelihood estimation and Promax rotation was fit. Linear regression was used to examine associations between extracted factor scores and psychosocial outcomes, such as overall mental and physical health, Post-Traumatic Stress Disorder (PTSD) symptom burden, and social participation.

**Results:**

We included 405 patients from 2015-2022, of whom: 66% were male; 82% were white; mean age was 45.4; median %Total Burn Surface Area (TBSA) was 14%; and 60.2% had a primary etiology of Fire/Flame. A 3-factor solution was identified based on eigenvalues and parallel analysis. Factor 1 (Itch-related Symptoms) explained 16% of the variance, with high loadings for itch interference (1.11) and moderate loadings for itch intensity (0.37). Factor 2 (Psychological Distress) explained 28% of the variance, with high loadings for anxiety (1.03) and depression (0.96) and moderate loadings for sleep disturbance (0.30). Factor 3 (Pain-related Symptoms) explained 22% of the variance, with high loadings for pain interference (1.00) and pain intensity (0.83) and moderate loadings on fatigue (0.60). %TBSA was the strongest predictor of higher scores on the itch and pain-related symptom factors and men had higher scores on the psychological distress factor (p <.05). The following significant associations with outcomes were identified: Higher pain-related factor scores were strongly associated with worse physical health, increased PTSD symptom burden, and reduced social participation; and higher psychological distress factor scores were strongly associated with worse mental health and increased PTSD symptom burden.

**Conclusions:**

Six months post-burn survivors’ symptoms appear to cluster into 3 factors: itch-related symptoms, psychological distress, and pain-related symptoms. Higher scores in pain and psychological distress clusters, but not itch, were significantly associated with worse physical, mental, and social function.

**Applicability of Research to Practice:**

These findings can help facilitate interventions for burn survivors by enabling more targeted and effective symptom management, focusing on the outcomes patients are most at risk for based on the symptom clusters in which they score highest.

**Funding for the Study:**

N/A